# Wild bitter gourd improves metabolic syndrome: A preliminary dietary supplementation trial

**DOI:** 10.1186/1475-2891-11-4

**Published:** 2012-01-13

**Authors:** Chung-Huang Tsai, Emily Chin-Fun Chen, Hsin-Sheng Tsay, Ching-jang Huang

**Affiliations:** 1 Department of Family Medicine, Cheng Ching Hospital, Taichung 407, Taiwan; 2 Graduate Institute of Biochemical Sciences and Technology, Chaoyang University of Technology, Wufong, Taichung 41349, Taiwan; 3 Department of Biochemical Science and Technology, National Taiwan University, Taipei 106, Taiwan; 4 Institute of Biomedical Nutrition, Hungkuang University, Sha Lu, Taichung 443 Taiwan

**Keywords:** Clinical trial, Metabolic syndrome, *Momordica charantia*, Supplementation, Waist circumference

## Abstract

**Background:**

Bitter gourd (*Momordica charantia *L.) is a common tropical vegetable that has been used in traditional or folk medicine to treat diabetes. Wild bitter gourd (WBG) ameliorated metabolic syndrome (MetS) in animal models. We aimed to preliminarily evaluate the effect of WBG supplementation on MetS in Taiwanese adults.

**Methods:**

A preliminary open-label uncontrolled supplementation trial was conducted in eligible fulfilled the diagnosis of MetS from May 2008 to April 2009. A total of 42 eligible (21 men and 21 women) with a mean age of 45.7 ± 11.4 years (23 to 63 years) were supplemented with 4.8 gram lyophilized WBG powder in capsules daily for three months and were checked for MetS at enrollment and follow-up monthly. After supplementation was ceased, the participants were continually checked for MetS monthly over an additional three-month period. MetS incidence rate were analyzed using repeated-measures generalized linear mixed models according to the intention-to-treat principle.

**Results:**

After adjusting for sex and age, the MetS incidence rate (standard error, *p *value) decreased by 7.1% (3.7%, 0.920), 9.5% (4.3%, 0.451), 19.0% (5.7%, 0.021), 16.7% (5.4%, 0.047), 11.9% (4.7%, 0.229) and 11.9% (4.7%, 0.229) at visit 2, 3, 4, 5, 6, and 7 compared to that at baseline (visit 1), respectively. The decrease in incidence rate was highest at the end of the three-month supplementation period and it was significantly different from that at baseline (*p *= 0.021). The difference remained significant at end of the 4th month (one month after the cessation of supplementation) (*p *= 0.047) but the effect diminished at the 5th and 6th months after baseline. The waist circumference also significantly decreased after the supplementation (*p *< 0.05). The WBG supplementation was generally well-tolerated.

**Conclusion:**

This is the first report to show that WBG improved MetS in human which provides a firm base for further randomized controlled trials to evaluate the efficacy of WBG supplementation.

## Introduction

Bitter gourd (*Momordica charantia *L.; BG) is a common tropical vegetable that has also been used in the traditional medicine. BG's anti-diabetic, anti-bacterial, antiviral and anticancer activities have been scientifically demonstrated in past decades [1,2]. Among these, the anti-diabetic activity and possible mechanisms of BG have been demonstrated in molecular, cellular and animal models as well as in human studies and extensively reviewed [3-5]. In addition, BG has been shown to ameliorate metabolic syndrome (MetS) in animal studies [6-8]. Nevertheless, it remains unclear if BG has beneficial effects on MetS in human.

MetS is a metabolic disorder characterized by the clustering of risk factors including: abdominal obesity, dyslipidemia, hypertension and insulin resistance, and is well-established for predicting high risks of type 2 diabetes mellitus (DM), cardiovascular diseases [9] and all causes mortality [10]. Due to rapid transitions toward excessive energy intake and sedentary lifestyle, MetS has become a major health problem worldwide affecting about 34% of US [11] and 23.1% of Taiwan populations [12].

Insulin resistance, which leads to derangements in carbohydrates and lipid metabolism, is considered to play a central role in MetS. Peroxisome proliferator-activated receptors (PPARs) are nuclear receptors that control lipid and carbohydrate metabolism. These receptors are regarded as important targets for treating MetS [13]. BG extract activated PPARα [14] and PPARγ [15,16], and thus were recognized as a PPARα/γ dual agonist [17]. In animal models, BG up-regulated PPARγ and PPARα-mediated pathways which is associated with improved MetS [6,18]. Together with other evidences that BG improved insulin signaling [8], BG is considered a potential "traditional Chinese medicines" in treating MetS [19].

To date, most published human clinical trials on BG were focused on the blood glucose control [20], very few were on the MetS. This study aimed at preliminarily exploring the potential effects of WBG on MetS. It is anticipated to learn from this preliminary study the safety, feasibility and validity of experimental design, including subject inclusion and exclusion criteria, dosage, period of supplementation, length of washout period, endpoint for examination, etc., to support double blind, randomized-placebo controlled clinical trials in the future.

## Materials and methods

### Materials

Fresh Hualien No. 4 wild bitter gourds (*Momordica charantia *L.; WBG) were developed, cultivated and provided by Hualien District Agricultural Research and Extension Station, Council of Agricultural, Executive Yuan, Taiwan. This specific hybrid strain was selected based on its high activity in the PPAR transactivation assay. A total of 502.8 kg fresh WBG (whole fruit including seeds) was lyophilized and pulverized. The dried WBG powder (27.4 kg) was filled into capsules (480 mg in each). The preparation of WBG capsules from fresh WBG was conducted by a GMP factory (Family Satisfied BIO-CHEM Co., Changhua, Taiwan) under our instruction and supervision.

### Subjects and Design

#### Study subjects

The study was approved by the Institutional Review Board (IRB) of Cheng Ching Hospital (Taichung, Taiwan). Candidates (≥20 years) were recruited at this hospital and screened. Eligible subjects were invited and informed of the purpose and risks of the study and their written informed consents were obtained.

#### Definition of MetS and Exclusion criteria

Candidates having three or more of the following five features met the diagnostic criteria for MetS: (1) waist circumference ≥90 cm in men and ≥80 cm in women, (2) serum triglyceride ≥150 mg/dL, (3) high density lipoprotein cholesterol (HDL-c) < 40 mg/dL in men and < 50 mg/dL in women, (4) blood pressure ≥130/85 mm Hg, and (5) serum glucose ≥100 mg/dL [9,21]. Major exclusion criteria included: serum alanine aminotransferase ≥100 IU/L (3 times the upper limit of normal value), total bilirubin ≥2.0 mg/dL, creatinine ≥2.0 mg/dL, glycosylated hemoglobin (HbA1c) levels ≥9.0%, women of childbearing potential not using adequate contraception [2], diagnosed myocardial infarction, unstable angina or cerebral vascular attack in the previous six months, receiving vigorous exercise training, weight control with diet or drug, presence of conditions affecting compliance (i.e., drug, alcohol abuse or psychiatric problems), and unwillingness to consent. Those with allergies to foods of the melon family and with known G6PDH deficiency [2] were also excluded.

#### Sample size estimation

The estimation of the sample size was based on a 15% decrease in the incidence rate of MetS. As such, the investigation had more than 80% power to detect any 15% decrease in the incidence rate of MetS status at a significance level of 0.05. The expected dropout rate was 5%. More than 45 eligible patients were targeted for recruitment.

#### Study Design

This preliminary trial was regarded as a phase II (exploratory efficacy)-like study using an open-label single-arm design to generate hypothesis, to determine the safety (adverse effect) and to provide justification for a large-scale multicenter clinical trial in the future. Based on our previous animal study and the study reported by Jayasooriya et al [22], 1% (w/w) WBG in the diet significantly improved hyperglycemia and lipid metabolism. The dose of supplementation in this study was set at 4.5-5.0 g WBG/day as Taiwanese 24-hour diets were estimated to weigh 450-500 g on a dry basis [23]. Studied subjects were instructed to take three, three and four capsules with their three daily meals without altering their usual dietary and exercise habit. The MetS risk factors were monitored monthly throughout the three successive months of supplementation. After the cessation of supplementation, MetS risk factors were continually followed up over an additional three-month period. Thus, a total of seven visits was needed to complete the study (visit 1 was the baseline before the supplementation started). At each visit, subjects were interviewed regarding compliance and adverse events. Physical examination and blood specimen collection were also performed.

### Endpoints

#### Efficacy

The primary efficacy endpoint (outcome measurement) was a decline in the % MetS incidence after the intervention, while the secondary efficacy endpoints were the 5 diagnostic criteria for MetS and the insulin resistance indicators including homeostasis model assessment (HOMA), logHOMA, quantitative insulin sensitivity check index (Quicki) and McAuley index. These indicators were calculated as the followings: HOMA = insulin (μU/mL) × 0.8 glucose (mg/dL); QUICKI = 1/[log insulin (μU/mL) + log glucose (mg/dL)]; McAuley Index = exp [2.63-0.28 ln insulin (μU/mL) -28.35 ln triglycerides (mg/dL)]. Decreases of Homa & log Homa as well as increases of QUICKI and McAuley index values indicate the amelioration of insulin resistance.

#### Safety

In human trials of BG, the most commonly reported side effects are abdominal pain and diarrhea [24]. Aside from adverse events, other surrogate laboratory safety endpoints monitored were serum creatinine, hepatic transaminases (alanine aminotransferase and aspartate aminotransferase), γ-glutamyl transferase, alkaline phosphatase, total bilirubin, sodium and potassium.

### Physical examination

Weight, height, waist circumference, and blood pressure were measured by trained nurses, who also took blood samples. Weight and height were measured to the nearest 0.1 kg and 0.1 cm, respectively. Body mass index (BMI) was calculated as body weight in kilograms divided by the square of height in meters. Waist circumference was measured midway between the costal margin and iliac crest to the nearest 0.1 cm. At each visit, the blood pressure was measured 3 times with manual mercury sphygmomanometer and the mean value was recorded.

### Blood specimen collection and analysis

Fasting blood specimens were collected after an eight-hour overnight fast. The blood samples were sent to the clinical laboratory of Cheng Ching Hospital for analysis within one hour. Anti-coagulated whole blood was used to determine erythrocyte count, leukocyte count, hemoglobin, and platelet count using a Cell Dyn^® ^3500 hematology AutoAnalyzer (Abbott Laboratories, Dallas, TX, US). Biochemical tests were performed using a Hitachi^® ^747 analyzer (Roche Diagnostics, Mannheim, Germany).

### Data Analysis

Significance of the change in the MetS incidence rate was analyzed by the repeated-measures generalized linear mixed model (GLMM). Those of the secondary efficacy endpoints which were continuous variables were analyzed by the linear mixed model (LMM). Irrespective of whether the study subject has completed the whole study or not, data of the 42 study subjects were all included in the statistical analyses based on the "intention-to-treat" principle to avoid bias. The *p *values set the significance level at 0.05 were one-sided. The IBM SPSS^® ^19.0 for Windows was utilized to perform all the statistical analyses.

## Results

### Study Subjects

Among 58 participants assessed for eligibility, 42 (72.4%) met the inclusion criteria of MetS and 16 (27.6%) were excluded including 10 not meeting inclusion criteria and 6 refusing to participate in this trial. Among the 42 eligible enrolled, 38 completed the trial. The reasons of withdrawals were: two went abroad and two discontinued because of dizziness and headache.

Of the 42 study subjects, 21 (50%) were men and 21 (50%) were women. The study subjects had a median age (inter-quartile range; IQR) and mean age (range) of 49 years (35-54), 45.7 ± 11.4 years (23-63) respectively. Test of normality of age with Shapiro-Wilk test showed that *p*-value is 0.096, indicating that these data are from a normally distributed population. Eighteen (42.9%), 18 (42.9%) and 6 (14.3%) with 3, 4 and 5 criteria of MetS respectively were noted. Among the 42 subjects, 40 (95.2%) had abdominal obesity (based on waist circumference), 14 (33.3%) were overweight (24 < BMI < 27 kg/m^2^) and 26 (61.9%) were obese (BMI≥27 kg/m^2^). The numbers of subjects with other MetS risk factors were 26 (61.9%) with serum glucose ≥100 mg/dL, 34 (81.0%) with serum triglyceride ≥150 mg/dL, 25 (59.5%) with low HDL-c and 36 (85.7%) with SBP ≥130 or DBP≥ 85 mmHg. Additional characteristics of the study subjects included: tobacco use 7 (16.7%), alcohol consumption 2 (4.8%), betel nut chewing 1 (2.4%) and exercise 14 (33.3%).

The baseline characteristics of the 42 subjects, stratified by gender, are presented in Table 1. The following characteristics were significantly different between genders: body height, HDL-c, alanine aminotransferase, aspartate aminotransferase, γ-glutamyl transferase, total bilirubin, creatinine, uric acid, homocysteine, testosterone, C-reactive protein, red blood cell, hemoglobin, platelet, HOMA and McAuley index. No significant differences were noted between the two groups in terms of age, body weight, body mass index, waist circumference, blood pressure, heart rate, fasting glucose, glycosylated hemoglobin A1c, triglyceride, low density lipoprotein cholesterol (LDL-c), cholesterol, alkaline phosphatase, insulin, fibrinogen, white blood cell, logHOMA and Quicki.

**Table 1:** Baseline characteristics and differences between genders (n = 42)

Variables	Men mean	**SEM**^**a**^	Women mean	**SEM**^**a**^	***p *value**^**b**^
Age (year)	46.9	2.7	44.4	2.3	0.487
Body height (cm)	170.1	1.6	160.0	1.2	0.000
Body weight (kg)	82.4	3.1	77.1	3.4	0.258
Body mass index (kg/m^2^)	28.4	0.7	30.0	1.1	0.225
Waist circumference (cm)	98.4	1.8	94.4	2.3	0.181
Systolic blood pressure (mm Hg)	140.0	3.3	137.7	3.7	0.631
Diastolic blood pressure (mm Hg)	93.5	3.2	88.2	2.3	0.187
Heart rate (beat/min)	75.1	2.5	77.7	2.2	0.448
Fasting sugar (mg/dL)	112.8	5.8	105.4	5.0	0.339
Hemoglobin A1c^c ^(%)	6.4	0.3	6.3	0.2	0.684
Triglyceride (mg/dL)	234.0	23.8	188.4	14.7	0.112
HDL-c^c ^(mg/dL)	41.5	1.7	49.3	2.1	0.006
LDL-c^c ^(mg/dL)	125.5	7.1	134.9	6.1	0.314
Total cholesterol (mg/dL)	195.2	7.7	204.9	6.2	0.333
Alanine aminotransferase (IU/L)	42.0	4.1	23.2	2.4	0.000
Aspartate aminotransferase (IU/L)	30.1	3.1	20.5	1.6	0.008
γ-Glutamyl transferase (IU/L)	54.4	8.5	29.8	3.1	0.012
Alkaline phosphatase (IU/L)	161.3	7.8	142.2	9.8	0.135
Total bilirubin (mg/dL)	0.7	0.05	0.6	0.06	0.019
Creatinine (μm/L)	1.1	0.03	0.8	0.02	0.000
Uric acid (mg/dL)	7.8	0.29	6.1	0.27	0.000
Insulin (μIU/mL)	19.4	4.7	16.5	2.0	0.566
Homocysteine (μmol/L)	13.6	0.7	11.5	0.6	0.025
Testosterone (ng/dL)	337.2	25.0	31.6	3.7	0.000
Fibrinogen (mg/dL)	434.5	20.7	469.8	27.0	0.308
C-reactive protein (mg/dL)	0.28	0.06	0.51	0.12	0.001
White blood cell (k/μL)	7.3	0.4	7.2	0.5	0.792
Red blood cell (m/μL)	5.2	0.12	4.7	0.07	0.002
Hemoglobin (g/dL)	15.3	0.2	13.7	0.2	0.000
Platelet (k/μL)	259.1	11.0	312.4	11.9	0.002
HOMA^c^	4.8	1.1	3.7	0.5	0.012
LogHOMA	0.56	0.07	0.51	0.05	0.096
Quicki^c^	0.32	0.01	0.32	0.01	0.176
McAuley index	0.55	0.03	0.59	0.03	0.043

^a^SEM indicates standard error of mean. ^b^Analyzed by t-test. ^c^Hemoglobin A1c indicates glycosylated hemoglobin A1c. HDL-c indicates high density lipoprotein cholesterol. LDL-c indicates low density lipoprotein cholesterol. HOMA indicates homeostasis model assessment. Quicki indicates quantitative insulin sensitivity check index.

### Effects of WBG on MetS

**Figure 1:**
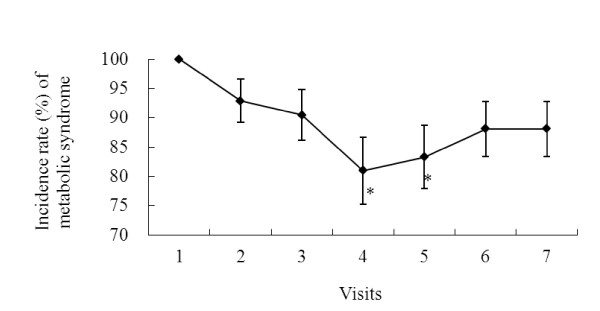
**Changes in the incidence rate of metabolic syndrome (MetS) throughout the study period**. 42 subjects were supplemented with 4.8 g/d wild bitter gourd for three month. Metabolic syndrome was monitored monthly during the three-month supplementation (visit 1-4) and another three months (visit 5-7) after the supplementation ended. *p < 0.05 denotes significantly different from the baseline value (visit 1) analyzed by generalized linear mixed models after adjusting for sex and age.

Changes in the incidence rate of MetS throughout the study are shown in Figure 1. The GLMM analyses were used to test the significance of these changes. After adjusting for sex and age, the decreases in the incidence rate (standard error, *p *value) of MetS at visit 2, 3, 4, 5, 6 and 7 from the baseline value (visit 1) were: 7.1% (3.7%, 0.920), 9.5% (4.3%, 0.451), 19.0% (5.7%, **0.021**), 16.7% (5.4%, **0.047**), 11.9% (4.7%, 0.229) and 11.9% (4.7%, 0.229) respectively. The incidence rate was lowest at the end of the three-month supplementation period (visit 4) and was significantly lower than that at the baseline which is 100% (*p *= 0.021). It still remained significant at visit 5 (one month after the cessation of supplementation) (*p *= 0.047) but this effect diminished at visit 6 & 7 (two & three months after stopping supplementation) (*p *> 0.05). The decreases were not significant when the two genders were analyzed separately.

Except for waist circumference, the remaining four risk factors of MetS did not show significant decreases after WBG supplementation. In contrast, significant decreases in the waist circumference were observed after the intervention (Table 2). Compared to the value at the baseline (visit 1), there were significant decreases in the waist circumference at visit 3 (*p *< 0.0001), visit 4 (*p *= 0.002) and visit 5 (*p *= 0.042).

**Table 2:** Changes of the waist circumference throughout the study period

Differences Between	Women			Men			Total		
	
	Mean (cm)	**SEM**^**a**^	***p *value**^**b**^	Mean (cm)	**SEM**^**a**^	***p *value**^**b**^	Mean (cm)	**SEM**^**a**^	***p *value**^**b**^
Visit 2-Visit 1^c^	0.13	0.88	0.878	-2.08	0.95	0.031	-0.99	0.66	0.138
Visit 3-Visit 1	-2.97	0.88	0.001	-2.04	0.95	0.034	-2.52	0.66	0.000
Visit 4-Visit 1	-2.50	0.86	0.004	-1.64	0.95	0.087	-2.09	0.66	0.002
Visit 5-Visit 1	-0.72	0.88	0.411	-1.96	0.95	0.042	-1.36	0.66	0.042
Visit 6-Visit 1	0.20	0.88	0.817	-2.69	0.95	0.006	-1.26	0.66	0.059
Visit 7-Visit 1	-0.08	0.89	0.925	-2.55	0.97	0.010	-1.32	0.67	0.051

^a^SEM indicates standard error of means. ^b^Analyzed by linear mixed models.

^c^Visit 1 is the baseline.

When women and men were separately analyzed after adjusting for age, the decreases of the waist circumference were still significant (Table 2). Compared to the baseline, there were significant decreases in the waist circumference for women at visit 3 and visit 4, but not at visit 6 and visit 7. In men, there were significant decreases at visit 2, 3, 5, 6 and 7. Interestingly, there were no significant decreases in BMI after WBG supplementation.

**Figure 2:**
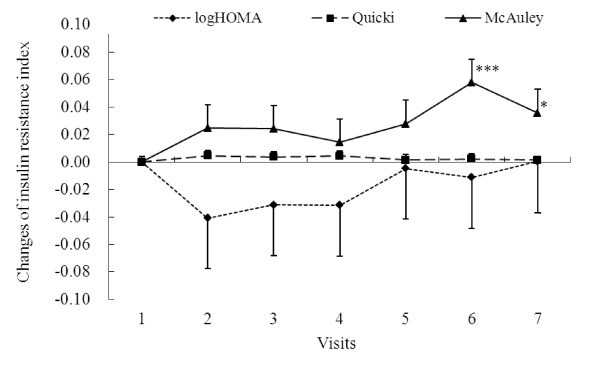
**Changes of logHOMA, Quicki and McAuley index, the insulin resistance indicators throughout the study period**. 42 subjects were supplemented with 4.8 g/d wild bitter gourd for three month. Insulin resistance index was monitored monthly during the three-month supplementation (visit 1-4) and another three months (visit 5-7) after the supplementation ceased. Values are means and error bars are 1/2 SEM. *p = 0.043 and ***p = 0.001 denote significantly different from the baseline value (visit 1) analyzed by linear mixed models after adjusting for age and sex.

Changes of the three insulin resistance indicators, logHOMA, Quicki, and McAuley values, throughout the study period are shown in Figure 2. The changes of insulin resistance indicators logHOMA, McAuley, and Quicki implied an ameliorative trend during the supplementation period. Decreased logHOMA and increased Quicki values showed improvement of insulin resistance at visit 2 and returned to nearly baseline level after the cessation of supplementation, but the differences did not reach significant levels (*p *> 0.05). The increasing trend of the McAuley values also suggested the improvement which could be maintained throughout the six month of the study period. The McAuley values were highest at visit 6 (two months after the cessation of supplementation) which was significantly different from that of visit 1. Result of LMM analyses, after adjusting for sex and age, showed the changes of the HOMA values at visit 2, 3, 4, 5, 6, and 7 from the baseline visit 1 were not significant (data not shown).

### Side effect

There were few adverse events during the study and these were generally mild. Throughout the three-month supplementation period only one subject reported abdominal pain and two claimed bloat. Neither upper respiratory tract infections, rapid weight gain, edema, nor other serious adverse events have been noted during the three-month follow-up period. Two patients dropped out due to persistent dizziness and headache which already disturbed them on and off before entering this study. There were no significant changes in the alkaline phosphatase, γ-glutamyl transferase, AST, ALT, total bilirubin, creatinine, sodium and potassium levels at visit 4 compared to those at the baseline (visit 1).

## Discussion

Results of this preliminary study demonstrate, for the first time, the beneficial effects of WBG in Taiwanese adult subjects with MetS. A daily dose of 4.8 grams lyophilized WBG powders in capsules significantly decreased the incidence of MetS after three months of supplementation and the improved status remained after stopping the supplementation for one month, but not for two and three months. This indicates that the washout period should be at least one month if a crossover study is to be conducted. Our results show that it is worth to conduct further randomized-placebo controlled trials to confirm the benefits of WBG on metabolic disorders.

MetS is a spectrum of metabolic disorders that project to high risk of type 2 DM and cardiovascular diseases. MetS subjects defined by at least three out of the five risk factors are thus a heterogeneous population. The most common characteristic of our study subjects is abdominal obesity, based on the large waist circumference (40 out of the 42 subjects). In this regard, we did observe a significant decrease in waist circumference after the supplementation of WBG. On the other hand, the number of subjects with high serum glucose(≥100 mg/dL) and triglyceride(≥150 mg/dL), low HDL-c and high blood pressure (SBP ≥130 or DBP≥ 85 mmHg) were 26 (61.9%), 34 (81.0%), 25 (59.5%) and 36 (85.7%), respectively. The relatively small sample size together with the heterogeneity might have limited our power to detect differences in these metabolic endpoints.

Insulin resistance is regarded to play a central role in MetS and abdominal obesity has been associated with insulin resistance. Despite that the insulin resistance indicators logHOMA, McAuley, and Quicki showed ameliorated trends after one to three months of WBG supplementation, there were no statistical significance due to the small sample size and heterogeneous characteristics of our study subjects. Indeed, our original goal was to recruit 45 MetS subjects based on the sample size estimation with a 15% decrease in the MetS incidence rate after WBG supplementation and 5% dropout rate. Fortunately, a 19% decrease in the MetS incidence rate was observed despite that only 42 subjects were recruited and 4 (9.5%) of them have dropped out.

In addition to the individual differences in MetS risk factors, the heterogeneity of our study subjects also arisen from the great range of their ages (23-63 years) and BMI (22.3-45.2). Moreover, both men and women (irrespective of whether they are post- or pre- menopause) were included. Besides, subjects previously diagnosed with type 2 DM, hyperlipidemia or hypertension were not excluded and were all advised to maintain their prescribed medication. These further increased the heterogeneity of our study subjects. All these heterogeneities should be avoided if a randomized controlled trial is to be carried out in the future.

The regulation of glucose metabolism by BG or it extracts/components has been extensively reviewed and various active compounds and action mechanisms have been proposed [3-5]. Early study isolated a Polypeptide-P, the so-called "plant insulin" from BG and demonstrated its insulin-like activity [25]. D-(+)-trehalose isolated from BG was shown to inhibit α-glucosidase [26] and delayed the digestion and absorption of dietary carbohydrates. Recently, cucurbitane-type triterpenoids isolated from BG were shown to increase glucose transporter 4 translocation through activation of AMPK [27], inhibit α-glucosidase [28], and modulate insulin secretion activity [29]. 9c,11t,13t-conjugated linolenic acid [30] and momordin [31] from BG were noted to activate PPAR α, γ and PPAR β/δ respectively. It has been shown that PPARγ agonist induced uncoupling protein-1 expression and increased oxygen consumption in the white adipose tissue cells [32]. In addition, PPARβ/δ has been shown to play a critical role in the body fat reduction in an animal model [33].

*In vivo *studies using animal models also provided evidences that BG ameliorated metabolic disorders through the regulations of PPARs as one of the mechanism [18,34]. The Hualien No.4 WBG used in this study is a hybrid variety of WBG being selected for its high PPAR activating activity. Other proposed mechanisms for actions of BG include stimulation of pancreatic insulin secretion [35], decreased hepatic gluconeogenesis, increased hepatic glycogen synthesis, metformin- and sulfonylurea-like glycemic control activities [5,36].

Almost all the human studies associated with metabolic disorder using bitter gourd were focused on controlling type 2 DM [3]. The beneficial effects of BG on type 2 DM have not been firmly established. Most non-controlled, and 2 randomized controlled trials [20,37] showed statistically significant reduction of fasting blood glucose, but other controlled trials showed no significant effect [20].

Among the five criteria of MetS, only waist circumference was found to be significantly reduced after supplementation in this study. This trend of change in the waist circumference was similar to the decline in the incidence rate of MetS, despite that lowering of waist circumference to less than 90/80 cm were not the main contributors for subjects that showed a release from having MetS after three months of BG supplementation. Abdominal obesity is well recognized as a potential etiologic factor of MetS [38]; hence, reverted central obesity is of significance for overall MetS management. This finding from our study is particularly important for Asians, a population that is likely to have metabolic obesity [39].

In general, lyophilized WBG powder capsule preparations in our study were well tolerated. There were few adverse events and these were generally mild. In previous human trials, the most commonly reported side effects are abdominal pain and diarrhea [24]. Our study showed that adverse effects were mostly moderate, including one and two subjects with abdominal pain and bloat respectively. To our knowledge, this is the first human trial to evaluate the effects of WBG on MetS.

### Limitation

Our study, however, has certain limitations. The relatively small sample size and substantial heterogeneity of the studied subjects limited our power to detect differences in many metabolic endpoints. This was an uncontrolled trial, so we cannot exclude the placebo effect. Nevertheless, after discontinuing the supplementation agent, our subjects were followed up for MetS over an additional three-month period. On the other hand, a recent study demonstrated that taking dietary supplements may create an illusory sense of invulnerability that disinhibits unhealthy behaviors because such supplements are perceived as conferring health advantages [40]. This might also be a concern as we did not monitor diets and other life-style factors of our subjects throughout the study period. Generalizability (external validity) of the trial findings were not defined due to the WBG capsules used were prepared from a specific variety of WBG provided by Hualien District Agricultural Research and Extension Station, Council of Agricultural, Executive Yuan, Taiwan.

## Conclusions

Results of this preliminary study demonstrate the beneficial effects of WBG on MetS in human. Daily dose of 4.8 g lyophilized WBG powder significantly decreased the MetS incidence rate after three months of supplementation and this improved status remained for one month, but not additional months, after the supplementation ceased. This indicates that the washout period should be at least one month if a crossover study is to be conducted. Waist circumference also decreased significantly after WBG supplementation. No major adverse effects were observed. Results of this study provide the justification for double-blinded placebo controlled trials in the future.

## List of abbreviations used

BG: bitter gourd; BMI: body mass index; DM: diabetes mellitus; HDL-c: high density lipoprotein cholesterol; GLMM: generalized linear mixed model; HOMA: homeostasis model assessment; LMM: linear mixed model; MetS: metabolic syndrome; PPAR: peroxisome proliferator-activated receptor; Quicki: quantitative insulin sensitivity check index; WBG: wild bitter gourd

## Competing interests

The authors declare that they have no competing interests.

## Authors' contributions

CHT wrote the manuscript, conducted the trial and statistical analysis and interpreted the data. ECFC coordinated the trial. HST and CjH provided critical review of the manuscript. CjH, ECFC and HST designed and supervised the study, obtained funding, interpreted the data, and provided critical review of the manuscript. All authors have read and approved the final manuscript.
